# Podocalyxin regulates pronephric glomerular development in zebrafish

**DOI:** 10.1002/phy2.74

**Published:** 2013-08-29

**Authors:** Koichiro Ichimura, Rebecca Powell, Tomomi Nakamura, Hidetake Kurihara, Tatsuo Sakai, Tomoko Obara

**Affiliations:** 1 Department of Cell Biology, University of Oklahoma Health Sciences CenterOklahoma City, Oklahoma; 2 Department of Anatomy and Life Structure, Juntendo University School of MedicineTokyo, Japan; 3 Department of Biological Science and Technology, Graduate School of Industrial Science and Technology, Tokyo University of ScienceNoda, Japan

**Keywords:** Glomerulogenesis, mucin domain, podocalyxin, podocytes, pronephros, zebrafish

## Abstract

Vertebrate glomerular podocytes possess a highly sialylated transmembrane glycoprotein, Podocalyxin. In mammals, the sialic acid of Podocalyxin plays a crucial role in the formation of the characteristic podocyte architecture required for glomerular filtration. We examined the function of Podocalyxin in the developing zebrafish pronephros by disrupting the expression of *podocalyxin* through the use of morpholino antisense oligonucleotides. Podocalyxin was localized at the apical membrane of podocytes throughout pronephric glomerular development in zebrafish. Translational blocking of *podocalyxin* expression resulted in pericardial edema and a hypoplastic glomerulus. Whereas regular foot processes with a slit diaphragm covered 66.7 ± 7.8% of the urinary surface of glomerular basement membrane in control fish, only 14.4 ± 7.5% of this area was covered with regular foot processes in the translationally blocked morphants. Splice blocking of *podocalyxin* exon 2, which partially encodes the bulky mucin domain with extensive sialic acid-containing sugar chains, resulted in the deletion of 53% of mucin domain-coding sequence from *podocalyxin* mRNA. Approximately 40% of these splice-blocked morphants had mild pericardial edema. Although the pronephric glomerulus in the splice-blocked morphants exhibited almost normal appearance with developed glomerular capillaries and mesangium, they had only 36.3 ± 6.9% of the area covered with regular foot processes. In conclusion, Podocalyxin is predominantly expressed in the podocytes and plays a distinct role in the formation of the podocyte foot processes with a slit diaphragm during zebrafish pronephric development.

## Introduction

The pronephros of small aquatic animals including zebrafish has been widely used to study kidney development and disease. It serves as a primary osmoregulatory organ in the larvae of teleost fish and amphibians (Howland 1916; Tytler et al. 1996). Teleost fish generally possess a pair of functional pronephroi consisting of three anatomical subunits: the glomerulus, pronephric tubule, and pronephric duct (Drummond et al. 1998).

The glomerulus exhibits a common structural organization among all taxonomic groups of vertebrates (Ichimura et al. 2007). Structurally, the glomerulus can be divided into vascular and epithelial regions. The vascular region is a core structure of the glomerulus and comprises the capillary loops and mesangium. This region is surrounded by the epithelial, a sheet-like structure consisting of the podocytes and the glomerular basement membrane (GBM). The vertebrate podocyte is an epithelial cell highly specialized for glomerular filtration. The basic cytoarchitecture of podocytes is also conserved throughout most vertebrates (Takahashi-Iwanaga 2002; Ichimura et al. 2007, 2009). It comprises three subcellular compartments: a cell body, major processes that extend outward from the cell body, and more distally located foot processes that are spanned by a slit diaphragm (Kriz and Kaissling 2007). Podocytes adhere to the GBM primarily via their numerous foot processes, and the cell body of podocytes thus does not directly attach to the GBM, but leaves a subpodocyte space between the cell body and filtration barrier (Neal et al. 2005).

The podocyte apical membrane is abundant in negatively charged sialic acid-rich glycoconjugates, which are detected by using cationic dyes and sialic acid-specific lectins (Mohos and Skoza 1969; Michael et al. 1970; Holthofer et al. 1981). Under some pathologic conditions including nephrotic syndrome, podocytes reorganize their interdigitating fine foot processes into the broader flat protrusions (Shirato 2002; Kriz et al. 2013). This alteration is linked to a decrement of the podocyte surface charge, as a similar structural alteration can be induced by neutralization of membrane surface charge (Seiler et al. 1977; Kerjaschki 1978) or by removal of the sialic acid (Andrews 1979).

Podocalyxin is an extensively sialylated type I transmembrane protein that likely accounts for the majority of podocyte surface charges (Kerjaschki et al. 1984). Podocalyxin is found at the podocyte surface membrane in a variety of vertebrates (Ichimura et al. 2007), suggesting that the function of Podocalyxin in the glomerulus is highly conserved in vertebrate phylogeny.

**Figure 1:**
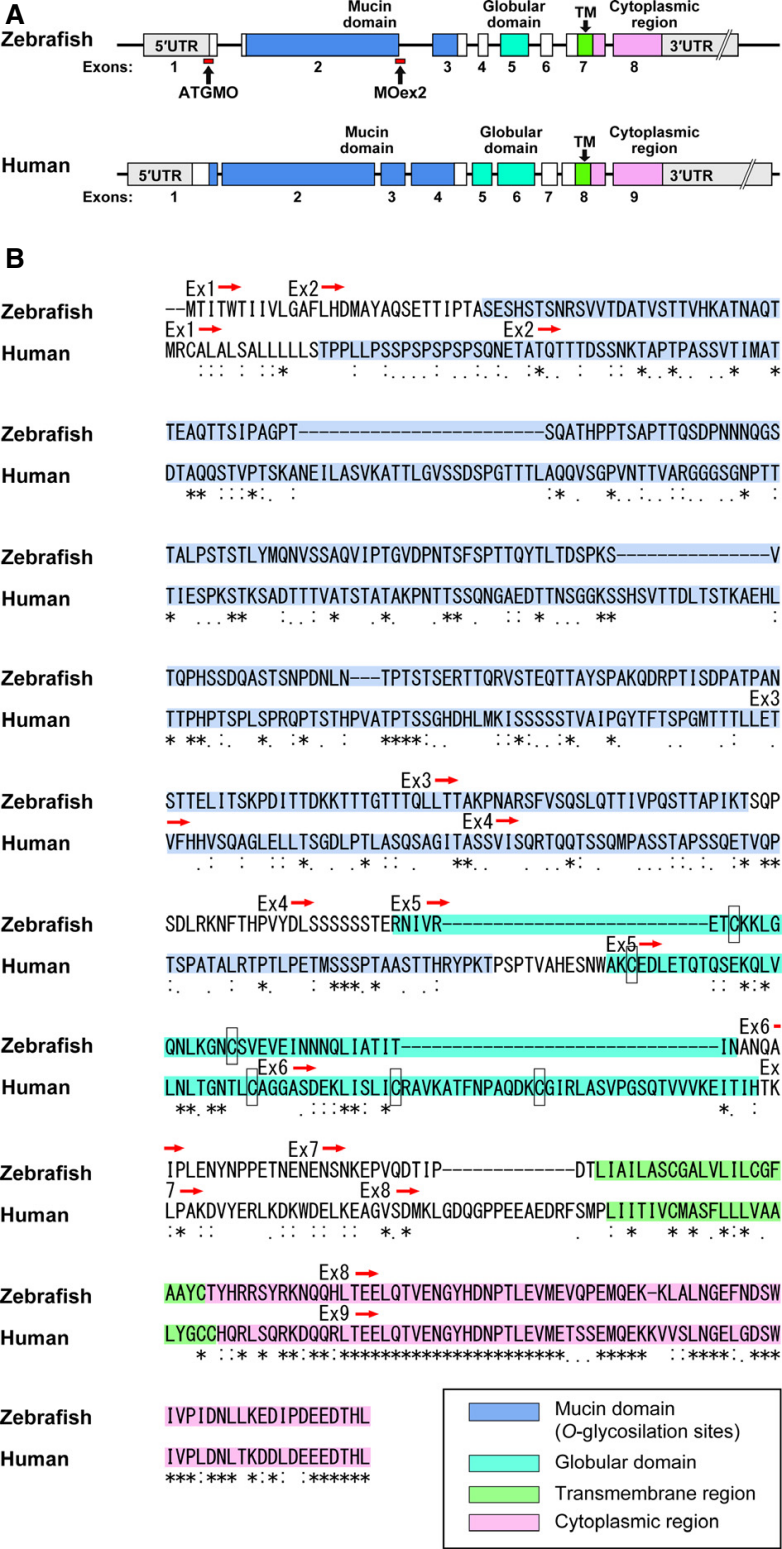
Genomic organization and amino acid sequence. (A) The zebrafish *podocalyxin* and human *PODOCALYXIN* genes contained eight and nine protein-coding exons, respectively. Red rectangles indicate the sites targeted by the morpholinos used. (B) ClustalW alignment of amino acid sequences. In both animals, the orthologues contain a bulky mucin domain (dark blue), a disulfide-bonded globular domain (bluegreen), a transmembrane region (green), and a cytoplasmic tail (pink), in this order. Cysteine residues in the globular domain are indicated by framed rectangles.

Podocalyxin in mammals comprises a bulky mucin domain, a disulfide-bonded globular domain, a transmembrane region, and a cytoplasmic tail (Fig. 1A) (Kershaw et al. 1995, 1997; Doyonnas et al. 2001). The mucin domain is extensively glycosylated and sialylated. Sialylation of Podocalyxin is crucial for the linkage between the cytoplasmic tail and cortical actin cytoskeleton (Orlando et al. 2001), which is localized beneath the podocyte surface membrane (Ichimura et al. 2003, 2009). The linkage between podocalyxin and the actin cytoskeleton is believed to maintain the structural integrity of the podocyte foot processes, because disruption of the linkage is associated with some experimental models of nephrotic syndrome (Takeda et al. 2001).

In this study, we examined the localization of Podocalyxin during the development of the zebrafish pronephric glomerulus and disrupted the expression of *podocalyxin* by using morpholino antisense oligonucleotides to study its role for the formation of the characteristic architecture of podocytes.

## Material and Methods

### Fish maintenance

The AB strain of zebrafish was maintained at 28.5°C under a 14-h light/10-h dark cycle. Embryos were kept at 28.5°C in 0.5 × E2 egg medium (7.5 mmol/L NaCl, 0.25 mmol/L KCl, 0.5 mmol/L CaCl_2_, 0.5 mmol/L MgSO_4_, 0.075 mmol/L KH_2_PO_4_, 0.025 mmol/L Na_2_HPO_4_, 0.35 mmol/L NaHCO_3_, 0.01% methylene blue) (Westerfield 2000). All animal experiments were performed in strict accordance with the recommendation in the Guide for the Care and Use of Laboratory Animals of the National Institutes of Health, and were covered by protocols approved from the Institutional Animal Care and Use Committee of the University of Oklahoma Health Sciences Center (IACUC protocol #12-033 to T. O.).

### Genomic structure and peptide motif analyses

Full-length cDNA sequences of zebrafish *podocalyxin* and human *PODOCALYXIN* were obtained from the Ensemble database (transcript ID: ENSDART00000102301 and ENST00000378555, respectively). Potential mucin-type *O*-linked glycosylation sites (mucin domain) and the transmembrane region were predicted by using the NetOGlyc (http://www.cbs.dtu.dk/services/NetOGlyc) (Julenius et al. 2005) and TMHMM (http://www.cbs.dtu.dk/services/TMHMM) (Krogh et al. 2001) servers, respectively.

### Cloning of zebrafish podocalyxin

Zebrafish full-length *podocalyxin* cDNA was obtained by using RT-polymerase chain reaction (PCR) on total RNA that was isolated from 4-dpf (days post fertilization) embryos using an RNAqueous-4PCR Kit (Invitrogen, Carlsbad, CA). RT-PCR was performed using the SuperScript III One-Step RT-PCR System with Platinum Taq High Fidelity (Invitrogen) followed by nested PCR using Phusion High-Fidelity DNA Polymerase (Finnzymes, Espoo, Finland). The primers used are as follows: 5′-AGC TGC AGA GCA AAC AAA AGC-3′ and 5′-TCT TGA CAT TCT GGC TGG TCA-3′ (RT-PCR); 5′-GAA GAG ACT GAA CGC GGA GAA-3′ and 5′-TTA CCG TAA AGG CAG CAG CAG-3′ (nested PCR). The full-length cDNA was subcloned into pCRII-Blunt-TOPO (Invitrogen), and verified by DNA sequencing.

### Morpholino antisense oligonucleotides and assessment of efficacy

Two independent morpholino antisense oligonucleotides were designed, including one that blocks translation of *podocalyxin* mRNA (*podxl*ATGMO: 5′-GGT CAT TTT CAG ATT CTC CGC GTT C-3′) and one that blocks splicing of *podocalyxin* exon 2 (*podxl*MOex2: 5′-CTG ATG TGA GCG AAA TCT TAC TTG T-3′) (Gene Tools, Philomath, OR). *podxl*MOex2 targets the splice acceptor site of the exon 2 (Fig. 1A). The morpholinos were diluted with the injection solution containing 100 mmol/L KCl and 10 mmol/L HEPES (pH 7.6) and were injected at final concentrations of 0.15 mmol/L (*podxl*ATGMO) and 0.25 mmol/L (*podxl*MOex2) into one- or two-cell stage embryos using a Nanoliter2000 microinjector (World Precision Instruments, Sarasota, FL). Injection volume was ∼4.6 nL. Translational blocking by *podxl*ATGMO was verified by immunohistochemistry using anti-Podocalyxin antibody. Splice blocking by *podxl*MOex2 was verified by RT-PCR and nested PCR. Total RNA was isolated from five embryos with an RNAqueous-4PCR Kit and was used as RT-PCR template. RT-PCR was performed using SuperScript III One-Step RT-PCR System with Platinum Taq High Fidelity followed by nested PCR using Phusion High-Fidelity DNA Polymerase. The primer sets used for the assessing efficacy of *podxl*MOex2 were as follows: 5′-ACG CGG AGA ATC TGA AAA TGA-3′ and 5′-ATG TTT TGA TTG GGG CAG TTG-3′ (for RT-PCR); 5′-AAA TGA CCA TCA CGT GGA CAA-3′ and 5′-CTA GCG TTT GGT TTT GCT GTG-3′ (for nested PCR). The altered PCR product was subcloned into pCRII-Blunt-TOPO and sequenced.

### In situ hybridization

The pCRII-Blunt-TOPO-*podocalyxin* digested with *XhoI* was used as a template for the digoxigenin-labeled antisense RNA probe. The probe was synthesized by using the SP6 RNA polymerase (New England BioLabs, Ipswich, MA) and the DIG-RNA labeling kit (Roche Diagnostics, Mannheim, Germany). The embryos were fixed in 4% PFA, 0.1% Tween 20 in PBS for 2 h at RT, changed to 100% methanol, and stored at −20°C. Whole mount in situ hybridization was performed as described previously (Thisse and Thisse 2008). Alkaline phosphatase-conjugated anti-digoxigenin (Roche Diagnostics) was used to localize the probes. Nitro-blue tetrazolium chloride (NBT)/5-Bromo-4-Chloro-3′-Indolylphosphatase *p*-Toluidine salt (BCIP) (Roche Diagnostics) was used as the chromogenic substrate to produce the blue staining. After color development, samples were dehydrated with a graded series of methanol and embedded in JB-4 resin (Polysciences, Warrington, PA). Ten micrometer-thick sections were cut by a RN2255 microtome (Leica Microsystems, Wetzlar, Germany) and counterstained with eosin II (BBC Biochemical, Mount Vernon, WA). After mounting in Poly-Mount (Polysciences), the stained sections were photographed using a Provis AX-70 microscope (Olympus, Tokyo, Japan) equipped with a RETIGA EXi digital camera (QImaging, Surrey, Canada).

### Antibodies

The rabbit polyclonal anti-rat podocalyxin antibody was raised against the glutathione S-transferase fusion protein with the entire cytoplasmic region of the rat podocalyxin (Kobayashi et al. 2009). The amino acid sequence of this region is highly conserved between zebrafish and mammals. This antibody was shown to delineate the podocyte surface membrane in other vertebrates including carp, bullfrog, newt, turtle, and gecko (Ichimura et al. 2007). Mouse monoclonal anti-rat ZO-1 (a kind gift from Drs. S. Tsukita and S. Tsukita) was raised against a fraction for adherence junctions obtained from rat livers (Itoh et al. 1991).

### Immunohistochemistry

Immunohistochemistry was conducted as previously described (Ichimura et al. 2013). In brief, embryos were fixed with Dent's fixative (20% DMSO in methanol) overnight at 4°C. Fixed samples were rehydrated with a graded series of methanol, and washed with PBS containing 0.5% Triton X-100 (PBSTx). Subsequently, the samples were blocked with incubation solution (PBSTx containing 10% normal goat serum, and 1% DMSO) for 2 h at RT, and incubated with the anti-podocalyxin antibody (working dilution 1:100) with the incubation solution for 10–15 h at 4°C. After washing with PBSTx, the samples were incubated with Alexa-Fluor546-conjugated goat anti-rabbit IgG (H+L) (Jackson ImmunoResearch Laboratories, West Grove, PA) diluted with the incubation solution (1:1000) for 2 h at RT. Some samples were subsequently incubated with anti-ZO-1 antibody with incubation buffer (1:100) for 10–15 h at 4°C, and then with Alexa-Fluor488-conjugated goat anti-mouse IgG (H+L) (Jackson ImmunoResearch Laboratories) with the incubation solution (1:1000) for 2 h at RT. Stained samples were dehydrated with a graded series of methanol, embedded in JB-4 resin, and cut into 10 μm-thick sections by a RN2255 microtome. After being mounted in anti-fading mounting medium (90 mL glycerol, 10 mL PBS, 100 mg *p*-phenylenediamine) (Platt and Michael 1983), the sections were imaged using an FV-1000 confocal laser scanning microscope (Olympus).

### Histological analysis

Larvae were fixed with histology fixative (1.5% glutaraldehyde, 4% paraformaldehyde, 3% sucrose in 0.1 mol/L phosphate buffer, pH 7.3) overnight at 4°C, dehydrated by a graded series of methanol, and embedded in JB-4 resin. Four micrometer-thick sections were cut and stained with Harris hematoxylin and special eosin II (BBC Biochemical). The stained sections were imaged using a Provis AX-70 microscope equipped with a RETIGA EXi digital camera.

### Transmission electron microscopy and morphometry

Larvae were fixed with histology fixative overnight at 4°C. The samples were immersed in 1% OsO_4_ in 0.1 mol/L PB for 1 h, dehydrated with a graded series of ethanol, and then embedded in Epon-Araldite resin (Electron Microscopy Sciences, Hatfield, PA). Ultrathin sections were stained with uranyl acetate and lead citrate and imaged on an H-7600 transmission electron microscope (Hitachi High Technologies, Tokyo, Japan) or a JEM-1230 (JEOL, Tokyo). To evaluate podocyte damage, we quantified the relative surface area of the GBM covered by regular foot processes, irregular-shaped processes, and cell body on the largest cross section of pronephric glomerulus obtained from three individual fish in each experimental group by the use of ImageJ. Values are presented as means ± SD. Differences were tested using Student's *t*-test, with *P* < 0.05 being considered significant.

## Results

### Protein structure of zebrafish Podocalyxin

Amino acid alignment indicated the functional domains of Podocalyxin orthologues were conserved between zebrafish and human (Fig. 1). The zebrafish *podocalyxin* gene contains eight protein-coding exons. Exons 2 and 3 encode a bulky mucin domain, which is predicted to be extensively glycosylated and sialylated. The middle third of exon 7 encodes a transmembrane region (Fig. 1). Identity and similarity of overall amino acid sequences between zebrafish and human Podocalyxin are low (26.4% and 37.0%, respectively), but the intracellular regions showed a high degree of identity and similarity (72.4% and 82.9%, respectively).

### *Podocalyxin* mRNA expression in the pronephric glomerulus

**Figure 2:**
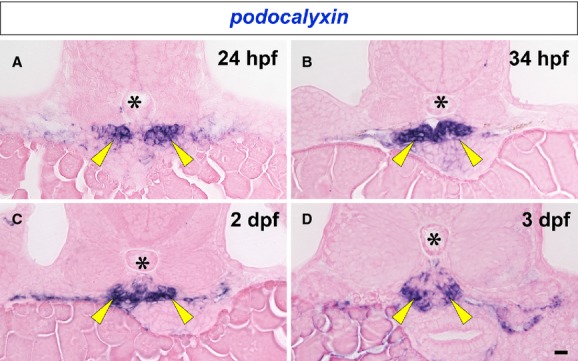
Expression of *podocalyxin* mRNA in developing pronephric glomerulus. *podocalyxin* mRNA is detected by in situ hybridization in the paired glomerular primordia at 24 and 34 hpf (arrowheads in A and B). Glomerular primordia fuse at the midline to form a glomerulus by 2 dpf, and *podocalyxin* expression continues in the glomerulus to 2 and 3 dpf (arrowheads in C and D). Asterisks indicate the notochord. Bar scale, 10 μm.

In situ hybridization for *podocalyxin* mRNA was performed on 24-hpf (hours post fertilization) to 3-dpf zebrafish. At 24 and 34 hpf, *podocalyxin* expression was already evident in the paired glomerular primordia, which comprised a pancake-shaped vesicular epithelium as shown in a previous report (O'Brien et al. 2011) (Fig. 2A and B). By 2 dpf glomerular primordia had fused with glomerular capillaries and mesangium at the midline to form a single glomerulus and *podocalyxin* expression continued in the glomerulus to 2 and 3 dpf (Fig. 2C and D).

### Localization of Podocalyxin in the developing pronephric glomerulus

**Figure 3:**
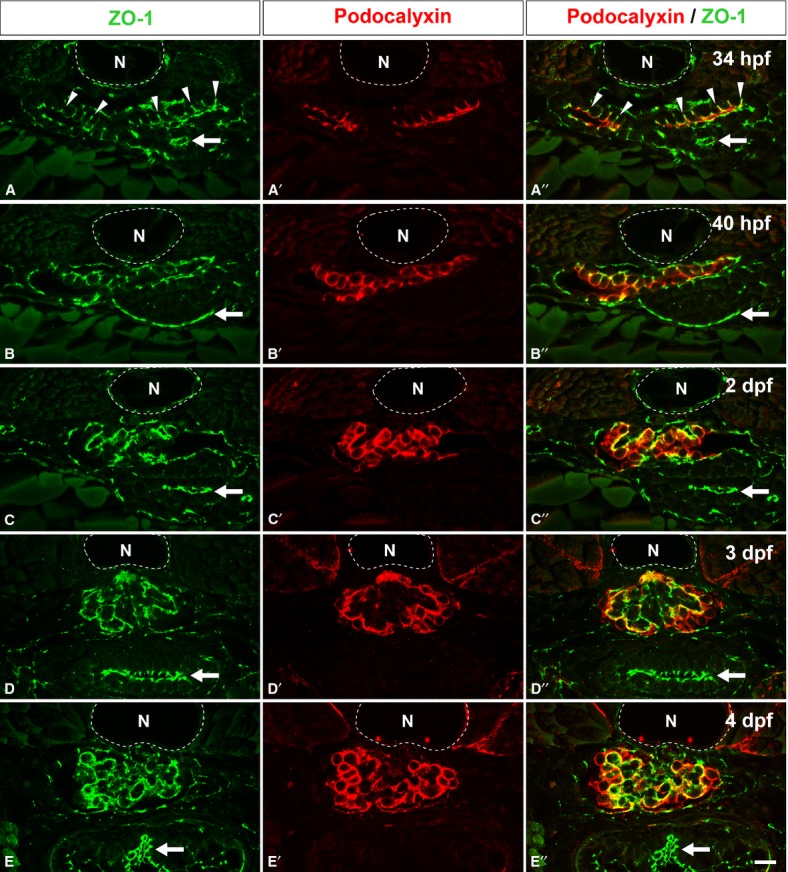
Localization of Podocalyxin in the developing pronephric glomerulus. Double immunofluorescence labeling for ZO-1 (green) and Podocalyxin (red) was performed to examine the localization of Podocalyxin in zebrafish at 34 hpf (A-A′′), 40 hpf (B-B′′), 2 dpf (C-C′′), 3 dpf (D-D′′), and 4 dpf (E-E′′). At 34 hpf, immunoreactivity for ZO-1 is found at the tight junctions between the primitive columnar podocytes (arrowheads in A, A′′). In addition, Podocalyxin is localized at the apical membrane, which is recognized as the luminal surface membrane above the tight junctions (A′, A′′). At 40 hpf, the Podocalyxin-localized apical membrane is expanded (B-B′′). After 2 dpf, immunoreactivity for ZO-1 is found along the glomerular wall (C, D, and E), which indicates that interdigitation between the podocytes has become extensively. The Podocalyxin signal is found along the entire surface of the podocyte cell bodies (C′-E′, C′′-E′′). (A′′-E′′) show the merged images. N, notochord; arrows, tight junctions in the intestinal epithelium. Bar scale, 10 μm.

To examine the localization of Podocalyxin in the developing glomerulus, we performed double immunolabeling for Podocalyxin and ZO-1, which is localized both at the intercellular junctions of presumptive podocytes and the slit diaphragm of mature podocytes (Schnabel et al. 1990). ZO-1 is a useful marker for evaluating the developmental stage of glomeruli in rodent metanephros (Ichimura et al. 2003). At 34 hpf, immunoreactivity for ZO-1 was found at the tight junctions between the primitive columnar podocytes, in a dot-like pattern (Fig. 3A). At the same stage, Podocalyxin was already clearly localized to the apical membrane, which can be recognized as the luminal surface membrane above the tight junctions (Fig. 3A′, A′′). The tight junctions then migrated toward the basal side of the cell, and the extent of Podocalyxin-enriched apical membrane was expanded by 40 hpf (Fig. 3B-B′′). After 2 dpf, immunoreactivity for ZO-1 was found along the GBM in a linear pattern (Fig. 3C–E), which indicated that interdigitation between the podocytes had occurred. Immunoreactivity for Podocalyxin was found along the entire surface of podocyte cell body (Fig. 3C′–E′, C′′–E′′).

### Translational blocking of *podocalyxin* caused hypoplasia of pronephric glomerulus

**Figure 4:**
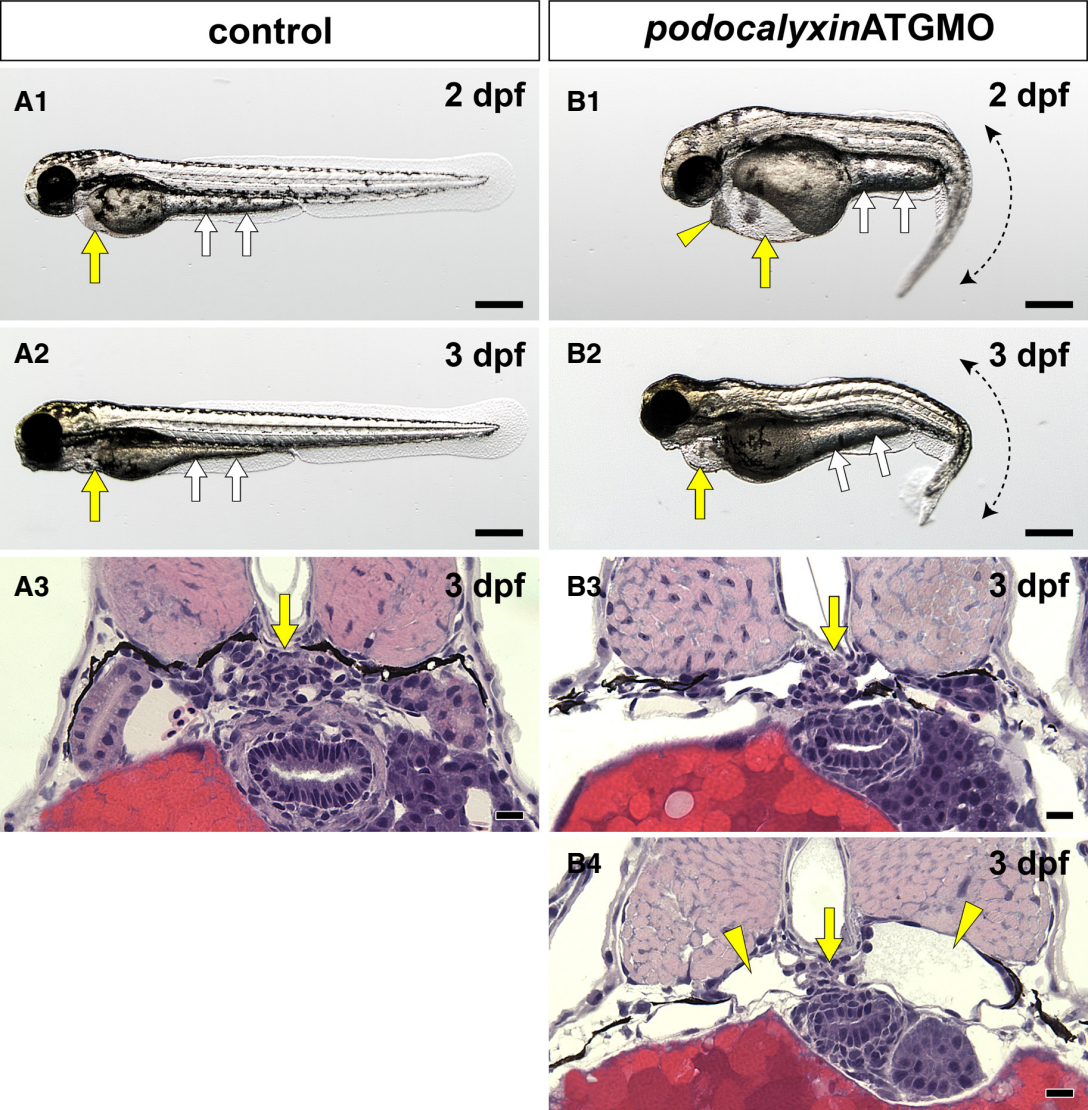
Translational blocking of *podocalyxin* caused pronephric glomerular hypoplasia. (A1, A2 and B1, B2) Whole-body images. Pericardial sac (yellow arrows) and yolk sac extension (white arrows) are larger in the *podxl*ATGMO morphants on 2 and 3 dpf (B1, B2) than in the uninjected control larvae (A1, A2). This enlargement resulted from edema. The *podxl*ATGMO morphants also have tail curvatures (B1, B2). In the 2-dpf morphants, cellular aggregation is consistently found at the anterior pole of pericardial sac (arrowhead in B1). (A3 and B3, B4) Hematoxylin–eosin staining sections showing pronephric glomerulus. In 3-dpf control larvae, a pair of pronephric nephron primordia merges to form a single glomerulus (arrow in A3). In the *podxl*ATGMO morphants, their pronephric glomeruli exhibit hypoplasia (arrows in B3, B4). Some of the morphants display bilateral cystic dilatation of Bowman's capsule and pronephric tubule (arrowheads in B4). Bar scales, 250 μm in A1, A2, B1, B2; 10 μm in A3, B3, B4.

To identify the function of Podocalyxin in pronephric glomerular development, we designed a translational blocking morpholino for *podocalyxin* (*podxl*ATGMO). The *podxl*ATGMO morphants consistently (∼95%) had a laterally curved tail and exhibited pericardial and yolk sac edema at 24 hpf, which persisted to 2 and 3 dpf (Fig. 4A1, A2 and B1, B2). We subsequently examined whether any morphological abnormalities were found in the pronephric glomerulus. In uninjected control zebrafish, a pair of pronephric nephron primordia merged with glomerular capillaries and mesangium at the midline to form a single glomerulus (Figs. 4A3, 5A1) (Drummond et al. 1998; Ichimura et al. 2012). In the *podxl*ATGMO morphants, a hypoplastic glomerulus was found beneath the notochord, and it was associated with dilated glomerular capillaries and poorly developed mesangium (Figs. 4B3, B4, 5B1). Approximately, 30% of the *podxl*ATGMO morphants had the bilateral cystic dilation in Bowman's capsule and the pronephric tubule (Fig. 4B4).

**Figure 5:**
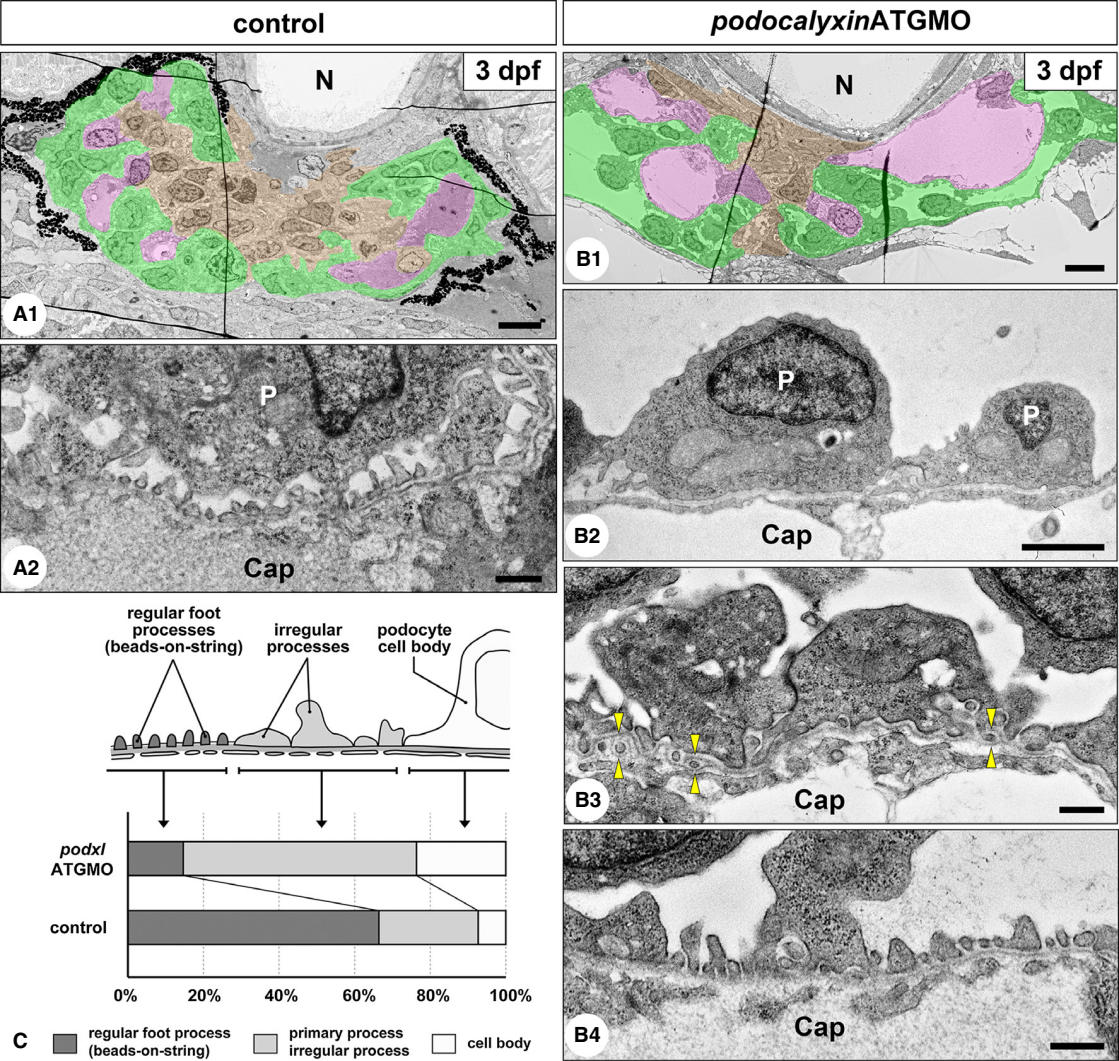
Translational blocking of *podocalyxin* caused hypoplasia of the podocyte foot processes. (A1–2) 3-dpf uninjected control embryos. The pronephric glomerulus contains several glomerular capillaries (pink) together with mesangium (brown). Bowman's capsule (podocytes and parietal epithelial cells) including urinary space is shown in green (A1). Podocytes form interdigitating fine foot processes with slit diaphragm, which exhibited a “beads-on-a-string” appearance. The cell body of podocyte is not in direct contact with the GBM to form a subpodocyte space under the cell body (A2). (B1–4) 3-dpf *podxl*ATGMO morphants. Glomerular capillaries vary in diameter, and poorly developed mesangium is found within the glomerulus (B1). Unlike in the control larvae, podocyte cell bodies directly adhere to the GBM (B2) and irregular-shaped processes covering the GBM (B3) are frequently found in the morphants, although regular foot processes are also formed in some regions (B4). Duplication of the GBM and the interposition of mesangial cell processes between the duplicated GBM are found in some regions (arrowheads in B3). (C) The ratio of the three structures covering the GBM. Cap, glomerular capillary lumen; N, notochord; P, podocyte cell body. Bar scales, 5 μm in A1, B1; 2 μm in B2, 500 nm in A2, B3, B4.

**Figure 6:**
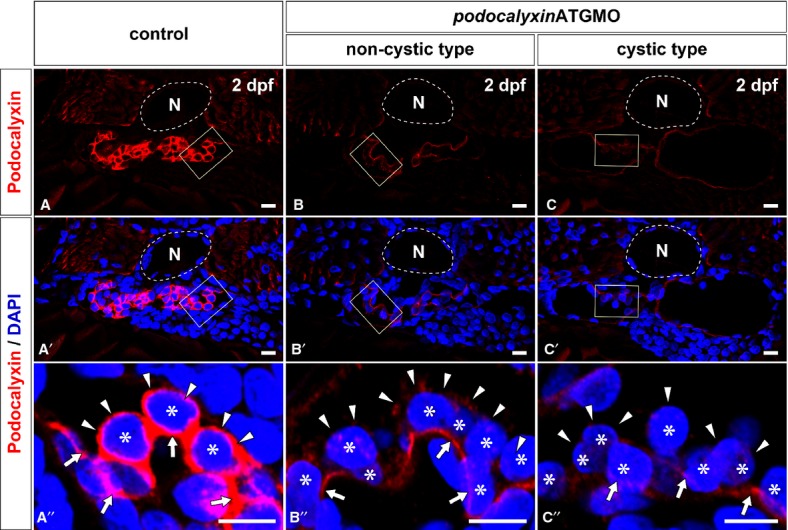
Efficacy for translational blocking of *podocalyxin*. The expression of Podocalyxin is determined by using immunohistochemistry with a specific antibody. Immunoreactivity for Podocalyxin is remarkably reduced in both the non-cystic type (B, B′) and the cystic type (C, C′) of glomerulus in 2-dpf *podxl*ATGMO morphants in comparison with control (A, A′). In control larva, immunoreactivity for Podocalyxin is recognized at the entire surface of podocyte cell body (arrowheads in A′′). Podocalyxin immunoreactivity surrounding the podocyte cell body is diminished in both the non-cystic and cystic types of glomeruli (arrowheads in B′′, C′′), although a faint signal is found in a linear pattern along the GBM (arrows in B′′, C′′). (A′′–C′′) images are magnification of the regions which are framed by rectangles in the above images. Asterisks indicate the nuclei of podocytes, which are visualized by staining with DAPI. N, notochord. Bar scales, 10 μm in A–C, A′–C′; 5 μm in A′′–C′′.

To test the efficacy of translational blocking, we examined the expression of Podocalyxin by using immunohistochemistry in non-cystic and cystic–type glomerulus on the 2-dpf morphants. Strong immunoreactivity for Podocalyxin in the control larvae was detected at the entire surface of podocyte cell body (Fig. 6A-A′′). On the other hand, Podocalyxin immunoreactivity was greatly diminished in both types of the *podxl*ATGMO morphant glomerulus, although a faint signal was found in a linear pattern along the GBM (Fig. 6B-B′′ and C-C′′).

Because *PODOCALYXIN*-deficient mice cannot form the regular foot processes and filtration slits at all, these mice have anuria and systemic edema (Doyonnas et al. 2001). We thus examined whether *podxl*ATGMO morphants have any ultrastructural abnormalities in the glomerular filtration barrier, especially foot processes and filtration slits. In 3-dpf control larvae, regular foot processes with a slit diaphragm, which occurred in a “beads-on-a-string” pattern, covered 66.7 ± 7.8% of the urinary surface of GBM (*N* = 3) (Fig. 5A2 and C). Other regions were covered with irregular-shaped processes (26.4 ± 5.7%) and podocyte cell body (6.8 ± 5.3%) (Fig. 5C). Most of the podocyte cell bodies were not in direct contact with the GBM, and the subpodocyte space was formed between the cell body and the filtration barrier. In the 3-dpf *podxl*ATGMO morphants which had non-cystic hypoplastic glomerulus, the area covered with regular foot processes was significantly smaller (14.4 ± 7.5%, *N* = 3) (Fig. 5B4 and C). Instead of regular foot processes, irregular-shaped processes (62.1 ± 10.7%) and podocyte cell bodies (23.5 ± 5.0%) covered the larger area of the GBM (Fig. 5B2, B3, and C).

In the 3-dpf control larvae, glomerular endothelial cells adhered to the GBM and possessed fenestrae (Fig. 5A2). In the 3-dpf *podxl*ATGMO morphants, glomerular endothelial cells and GBM exhibited almost same appearance found in the control (Fig. 5B4), although the duplicated GBM and the interposition of mesangial cell processes between the duplicated GBM were found in some regions (Fig. 5B3).

### Splice blocking of *podocalyxin* exon 2 encoding the mucin domain

**Figure 7:**
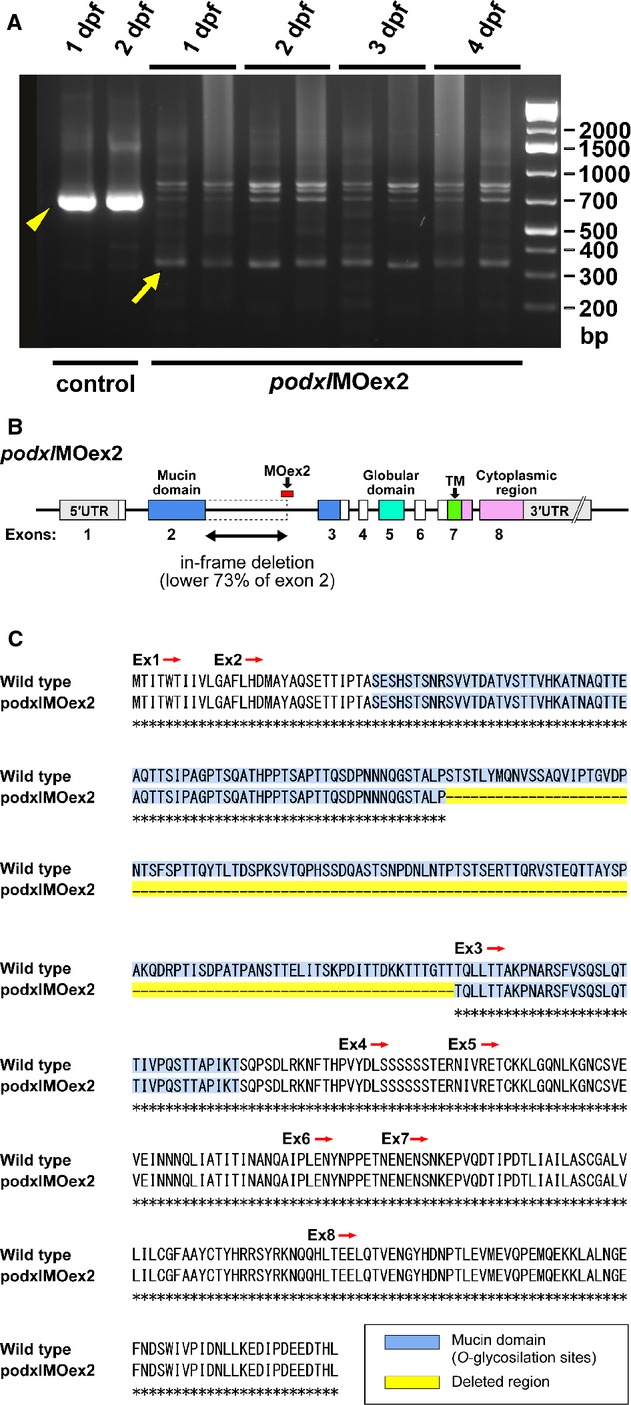
Efficacy of the morpholino antisense oligonucleotide targeting the splice acceptor site of *podocalyxin* exon 2 (*podxl*MOex2). (A) *podxl*MOex2 caused missplicing of the mRNA as detected by the presence of the altered PCR product, which is ∼360 bp smaller (arrow) than the wild-type product (arrowhead). The altered PCR product was present from 1 to 4 dpf. (B and C) Sequencing of the altered product revealed an in-frame deletion of lower 363 bp (73%) from exon 2, resulting in the deletion of 53% of the mucin domain from Podocalyxin in the *podxl*MOex2 morphants.

Podocalyxin has a bulky mucin domain, which is extensively glycosylated. The sialylation of these sugar chain termini is known to be crucial for the function of this protein in human and rodents (Seiler et al. 1977; Kerjaschki 1978; Andrews 1979; Takeda et al. 2001). In zebrafish, exon 2 of *podocalyxin* encodes 84% of the mucin domain and exon 3 encodes the rest (Fig. 1B). To examine the developmental function of this domain in zebrafish, we designed a splice blocking morpholino to target the splice acceptor site of *podocalyxin* exon 2 (*podxl*MOex2) (Fig. 7B).

**Figure 8:**
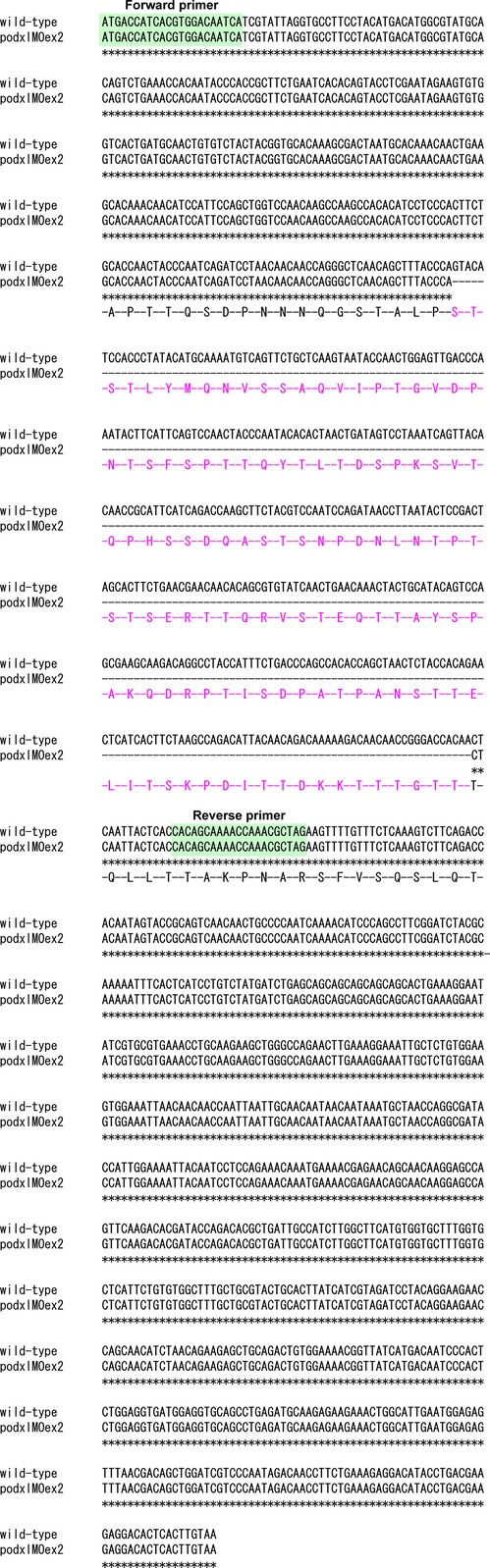
Partial in-frame deletion of *podocalyxin* exon 2 induced by *podxl*MOex2. Primers using efficacy checking (shown in Fig. 7A) were designed for highlighted regions.

Injection of *podxl*MOex2 resulted in misspliced mRNA as detected by an altered RT-PCR product, and the efficacy of knockdown continued until 4 dpf (Fig. 7A). This altered product was ∼360 bp smaller than the wild-type product. Sequencing of the altered RT-PCR product revealed an in-frame deletion of the lower 363 bp from exon 2 (Fig. 8), resulting in the deletion of 53% of the mucin domain-coding sequence from *podocalyxin* mRNA in the *podxl*MOex2 morphants (Fig. 7B and C). The resultant protein is predicted to have lost 121 amino acids encoded by exon 2, but all other domains of Podocalyxin will be preserved.

**Figure 9:**
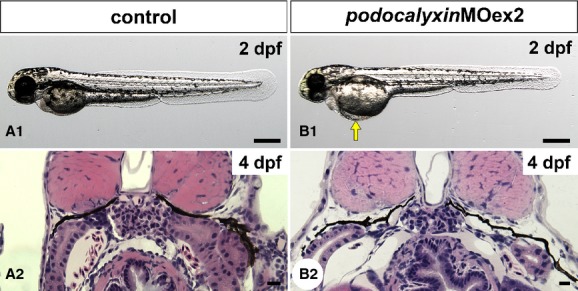
Truncation of *podocalyxin* exon 2 induces mild pericardial edema. (A1 and B1) Whole-body images of 2-dpf larvae. Approximately 40% of *podxl*MOex2 morphants have mild pericardial edema (arrow in B1). (A2 and B2) Hematoxylin–eosin-stained sections reveal that the pronephric glomerulus in the *podxl*MOex2 exhibits developed glomerular capillary and mesangium at 4 dpf (B2), as that seen in control (A2). Bar scales: 250 μm in A1, B1; 10 μm in A2, B2.

**Figure 10:**
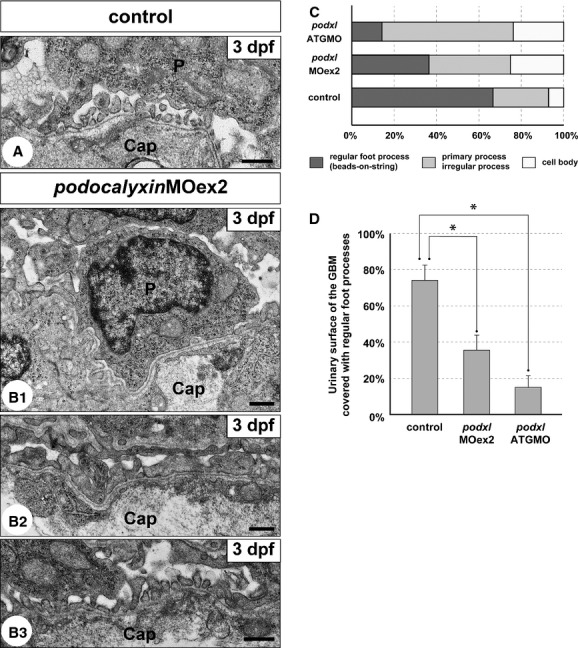
Truncation of *podocalyxin* exon 2 induces poor development of podocyte foot processes. (A and B1–3) Transmission electron micrographs showing glomerular capillary wall. Unlike control larvae (A), podocyte cell bodies that directly adhere to the GBM (B1) and irregular-shaped processes that cover the GBM (B2) are frequently found in the *podxl*MOex2 morphants, although regular foot processes with a “beads-on-a-string” pattern are also formed in some regions (B3). Cap, glomerular capillary lumen; P, podocyte cell body. Bar scales: 500 nm. (C) The ratio of the three structures covering the urinary surface of GBM. (D) Significant reduction in regular foot processes with slit diaphragm in the *podxl*ATGMO and *podxl*MOex2 morphants. In control larvae, regular foot processes with slit diaphragm cover 66.7 ± 7.8% of the urinary surface of the GBM (*N* = 3). The foot process-covering area is greatly reduced in the *podxl*MOex2 morphants (36.3 ± 5.0%, *N* = 3) and non-cystic-type *podxl*ATGMO morphants (14.4 ± 7.5%, *N* = 3) with statistical significance. All of the larvae were examined on 3 dpf. *P < 0.01.

Approximately 40% of the *podxl*MOex2 morphants had pericardial edema from 2 dpf (Fig. 9A1 and B1); however, this edema was much milder than that in *podxl*ATGMO morphants. In spite of the presence of pericardial edema, the pronephric glomeruli in the *podxl*MOex2 morphants were almost normal in appearance with well-developed glomerular capillaries and mesangium at the light microscopy level (Fig. 9A2 and B2). The *podxl*MOex2 morphants had significantly smaller areas (36.3 ± 6.9%, *N* = 3) covered with regular foot processes, in comparison with the uninjected control (Fig. 10A, B3, C and D). Instead of regular foot processes, irregular-shaped processes (38.6 ± 9.2%) and podocyte cell bodies (25.1 ± 3.6%) covered a larger area of the GBM (Fig. 10B1, B2, and C).

In the 3-dpf *podxl*MOex2 morphants, glomerular endothelial cells adhered to the GBM and possessed fenestrae, as found in control (Fig. 10A and B1–3). Unlike *podxl*ATGMO morphants, *podxl*MOex2 morphants did not exhibit the duplication of GBM.

## Discussion

### Podocalyxin expression in glomerular podocytes

Podocalyxin is a protein found at the surface membrane of pronephric, mesonephric, and metanephric glomerular podocytes in a variety of vertebrate classes including teleostei, amphibian, reptilian, Aves, and mammal (Ichimura et al. 2007, 2012). These facts strongly suggest that the function of Podocalyxin in the glomerulus is highly conserved in vertebrate phylogeny. Moreover, the mRNA of *podocalyxin* as well as those of *wt1a*, and *nephrin* in zebrafish have been expressed from the early phase before the vesicular glomerular primordia form (O'Brien et al. 2011; Ichimura et al. 2013), indicating that Podocalyxin is likely to play a role in the formation of pronephric glomerular primordia with other early phase-expressing factors.

### Similarities and differences in phenotypes observed in *podxl*ATGMO morphants and *PODOCALYXIN*-deficient mice

*PODOCALYXIN*-deficient mice die within 24 h of birth with anuric renal failure, which arises due to the failure of the podocytes to form the regular foot processes with slit diaphragm. Instead of the regular foot processes, podocyte cell bodies directly adhere to the GBM and completely engulf the vasculature (Doyonnas et al. 2001). These findings directly indicate that PODOCALYXIN plays a crucial role in the formation of the fine foot processes associated with slit diaphragm in mammals.

In *podxl*ATGMO morphants, fine foot processes with slit diaphragm were poorly developed and the final maturation failed to occur, indicating that Podocalyxin also has an important function for the proper formation of foot processes in zebrafish. Our data suggest that glomerular filtration occurred to some degree in these morphants; however, the persistence of severe pericardial and yolk sac edema meant that the ability of glomerular filtration was not capable of handling the volume that is necessary for the excretion of excess water. Unlike *PODOCALYXIN*-deficient mice, complete loss of foot processes, and slit diaphragm did not occur in the *podxl*ATGMO morphants. Protein expression of Podocalyxin was largely suppressed in the *podxl*ATGMO morphants, but not completely, resulting in the phenotypic difference in podocyte morphology between the knockout mice and the zebrafish morphants.

### Function of the highly sialylated mucin domain of Podocalyxin in the zebrafish pronephric podocytes

The podocyte surface membrane is abundant in negatively charged sialylated glycocalyx. At least PODOCALYXIN and NEPHRIN are highly sialylated and their sialylation is believed to be crucial to form and maintain the unique cytoarchitecture of podocytes (Kerjaschki et al. 1984; Dekan et al. 1991; Holzman et al. 1999; Weinhold et al. 2012). Several kinds of enzymes are involved in the synthesis and addition of sialic acid, and mutations in the key enzymes of the pathway in mice result in postnatal lethality due to podocyte disorder (Galeano et al. 2007; Weinhold et al. 2012). Uridine diphospho-*N*-acetylglucosamine 2-epimerase/*N*-acetylmannosamine kinase (GEN/MNK) is a bifunctional enzyme that synthesizes sialic acid, and *GEN*^*M712T/M712T*^ knock-in mice have a reduction in the sialylation level of PODOCALYXIN and cannot form podocyte foot processes and the slit diaphragm (Galeano et al. 2007). CMP-sialic acid synthetase (CMAS) is another key enzyme for the sialylation of sugar chains. Inactivation of the nuclear localization signal in CMAS using a knock-in strategy results in the down-regulation of CMAS and a subsequent reduction in sialylation for both PODOCALYXIN and NEPHRIN (Weinhold et al. 2012).

General depletion of sialylation affects the podocyte cytoarchitecture and glomerular filtration function as above summarized, but the specific depletion or reduction in sialylation in one kind of protein has not yet been elucidated. To begin to address this issue, we performed splice acceptor blocking of a mucin domain-coding exon in *podocalyxin* using *podxl*MOex2. In the *podxl*MOex2 morphants, 73% of podocalyxin exon 2 was skipped from the mRNA presumably resulting in the deletion of 53% of the mucin domain. The morphants exhibited mild pericardial edema and a significant reduction in regular foot processes with slit diaphragm, although both phenotypes were milder in the *podxl*MOex2 morphant than in the *podxl*ATGMO morphant. Thus, our data indicate that the bulky mucin domain through the highly sialylated sugar chains plays an important role in the formation of podocyte foot processes in the zebrafish pronephric glomerulus.

## Conclusion

Podocalyxin is predominantly expressed in podocytes throughout the zebrafish pronephrogenesis, and plays a distinct role in the formation of the characteristic cytoarchitecture of podocytes.
